# On the Basis of the Eau Medicinale D’Husson

**Published:** 1852-07

**Authors:** Thomas Bushell


### RECORD OF MEDICAL SCIENCE.

### MATERIA MEDICA AND THERAPEUTICS.

*On the Basis of the Eau Medicinale D'Husson.* By THOMAS BUSHELL, ESQ.—The subject which I am about to bring under the notice of the Pharmaceutical Society relates to the history of a very powerful article of the Materia Medica, the "EAU MEDICINALE D'HUSSON," which, although now obsolete, is still frequently referred to as the form in which colchicum was first introduced into medical practice; and, I trust, I shall be excused for occupying the attention of the meeting for a few minutes in relating some circumstances contributing to establish the identity of the *Colchicum Autumnale* as the basis of that remedy which enjoyed so great a repute about half a century ago as a proprietary specific for the treatment of gout and rheumatism.

A few days since I made inquiry of one of the chief vendors of patent medicines, at the west end of the town, if they now sold the Eau Medicinale; I was informed that it had not been inquired for for many years, but that they had been accustomed, when it was in demand, to receive it from a foreign perfumer in Bond Street. I then called at this house, and was politely informed that they were the proprietors and vendors of the Eau Medicinale D'Husson, but that they had not sold any for years, and that it had gone quite out of date. About four or five years ago they sold three or four bottles. I requested to be allowed to examine the remedy, and to see in what way it was sold or made up. After a little time two or three bottles were discovered; they held about two drachms, and were simply labelled "Eau Medicinale D'Husson." Printed wrappers, descriptive of its powers, were not at hand, and the parties seemed to have quite forgotten the doses in which it was administered. They informed me that the price was now nine shillings and sixpence, but was originally twenty-two shillings. The bottles were short and squat, quite curiosities in their way, and I could not resist revolving in my mind the great alteration which time and circumstances had made in the repute and consequent sale of this remedy, which at one time must have realized a splendid income to its proprietor.

The earliest medical notice I can find of the Eau Medicinale, is by Dr. Jones, in the year 1810, who had been on the continent in the years 1802 and 1803, with a gentleman who was a great sufferer from gout, and had then heard of the remedy of Husson, and which, on his return home in the year 1805, he was induced to make use of. He derived so much benefit from it, that from this time it made great advances as a remedy in England, and, to quote the words of the editor of the *Edinburgh Medical and Surgical Journal*, "noblemen and philosophers concurred in sounding its praises, if not in dancing hornpipes, in testimony of the new agility and flexibility of toe with which it had endowed them; and the President of the Royal Society, Sir Joseph Banks, who experienced the most extraordinary deliverance from his arch enemy, is said to have made it almost his pocket companion." It now became a great desideratum to ascertain its composition. Rhododendron, Chrysanthemum, Digitalis purpurea and lutea, Tobacco, Elaterium with opium, and many other plants, supposed to be analogous in their action to the magic water, were stated to form its basis.

Dr. Pereira states in his *Materia Medica* :

"The circumstances which have led to the extensive employment of colchicum in gout, are the following: About seventy years ago, M. Husson, a military officer in the service of the king of France, discovered, as he informs us, a plant possessed of extraordinary virtues in the cure of various diseases. From this plant he prepared a remedy called Eau Medicinale, which acquired great celebrity for abating the pain, and cutting short the paroxysm of gout. Various attempts were made to discover the nature of its active principle. In 1782, Messrs. Cadet and Parmentier declared that it contained no metallic or mineral substance, and that it was a vinous infusion of some bitter plant or plants. Alyon asserted that it was prepared with gratiola; Mr. Moore that it was a vinous infusion of white hellebore with laudanum; Mr. Want that it was a vinous infusion of colchicum. Although most writers have adopted Mr. Want's opinion, we should bear in mind that the proofs hitherto offered of its correctness, viz., analogy of effect, cannot be admitted to be conclusive, as is well shown by the fact that they have been advanced in favor of the identity of other medicines with the Eau Medicinale."

I should apologize for making so long an extract from Dr. Pereira's work, but as it contains a perfect epitome of all I have ever seen on the subject of the so-called gout specific, and as it leaves the question of the identity of its basis open to further inquiry, I hope that the circumstantial evidence which I now propose to lay before the Society, may somewhat clear up the point.

Mr. Want published his opinion in the *Medical and Physical Journal*, vol. xxxii., in the year 1814; and it was two or three years after that time, at the commencement of my apprenticeship, I was directed to prepare a preparation of colchicum in rum (four ounces of the dried cormus to a pint.) I was residing close to Covent Garden Market, and frequently had conversations on plants, &c., with the late Mr. Grimley. the herbalist, to whom I applied for the colchicum. Mr. Grimley inquired of me if I knew how it was that the use of this remedy was discovered by Mr. Want? He then related the following circumstance: —Mr. Want had returned home from visiting patients, and met his wife on the stairs, and told her that her father had been seized with a violent attack of the gout. The nursemaid, who was standing by, stated that she once lived with a little French gentleman, who prepared a secret remedy for the cure of the gout, the Eau Medicinale; that he was accustomed to carry on his operations at the upper part of the house; and that there was a door on the staircase which he always kept locked, but that she had managed to obtain a little bit of one of the articles he made use of, and that she then had it by her locked up in her box. Mr. W. had not much difficulty in gaining a sight of this treasure. It was unknown to him, but he at once proceeded to Covent Garden Market, and applied to my informant, Mr. Grimley, to ascertain if he could recognize the portion of bulb. Mr. G. pronounced it colchicum; and, to Mr. Want's further inquiry, informed him, that the only person who ever applied to them for it was a little Frenchman, and that he was accustomed to order from a cwt. to a cwt. and a half more than once in the season; that he adopted great precaution to prevent their obtaining a knowledge of what he did with it and from whence he came; that he always made inquiry if they had it ready, paid the amount charged, then brought a porter, who carried it to a hackney-coach which he had in waiting, and which immediately drove off with its precious charge—for precious it was, only to be compared to a lump of the Californian or Australian mineral, as its preparation sold for its weight in gold (two drachms twenty-two shillings.) This relation, although occurring about three or four and thirty years ago, is quite fresh in my memory. I have related it to many, and about seven years ago I was in attendance on the late Earl Thanet, who suffered much from gout, and had a great objection to take colchicum. I happened to mention this little history, when he said, "You are quite right." I met that little Frenchman repeatedly years ago at the late Duke of Queensberry's, who was a martyr to gout, and to whom he used to administer the Eau Medicinale, he being the preparer of it. In the course of inquiry I have also found good reason to suppose that this little Frenchman was a well-known foreign perfumer, and has been dead upwards of twenty years, the founder of the house who now profess to be the proprietors of this once celebrated remedy.

I have stated that it was in the thirty-second volume of the *Medical and Physical Journal*, that Mr. Want first made public his discovery of the basis of the Eau Medicinale D'Husson, but I find on further reference that it was not in a medical but in a popular journal, the *Monthly*, that he had previously announced it. I should take up too much of the time of the Society, if I entered into the controversies which took place at this period, with respect to the reliance which could be placed upon this beyond the numerous other drugs which had been as confidently declared to form its active ingredient, and it was the opinion of many that ere long it would be consigned to the tomb of the Capulets. I find, also, that a charge was made against Mr. Want, by an anonymous correspondent, that he had gained a knowledge of its basis by means somewhat similar to what I have now related, but which he denied, stating that he had obtained the first hint from Alexander, of Tralles, who recommended a remedy, "Hermodactylon," for the cure of gout, and with the effects of which the Colchicum Autumnale entirely corresponded; and that this plant had entered into many gout remedies that had become obsolete. All I am now desirous to say on this subject is, that the blue chamber being once penetrated, its mysteries might be readily compared with those which had preceded, but I believe the key was obtained in the manner I have related to the Society.

The present holders of the recipe for the Eau Medicinale, were so ignorant of its powers, as to inform me that two or three drops were a dose. I find that the usual dose was half a bottle, to be repeated, if the effect was not apparent, in four or six hours, but it was not uncommon for parties accustomed to its use, to take the whole two drachms, and repeated cases are on record of its powerful and dangerous effects; even life in some instances fell a sacrifice to its imprudent exhibition; and such was the experience also with the Colchicum Autumnale when first brought into practice, as it was considered to be a specific, and administered accordingly. This is not the place for entering into the therapeutical action of this valuable and potent plant, but its botanical and chemical characters are highly deserving the attention of the Members, and, combined with its Pharmaceutical preparations, might form matter for most interesting discussion.

There is a galenical compound in one of the French codexes called Eau Colchique D'Husson, prepared as follows:—Dry Colchicum, 60; Sherry, 125; twenty drops in a glass of *eau sucree* in gout and rheumatism; and there is a remark that this will be observed to differ from the tincture or anti-gout drops of Want, which has been given as the Eau Medicinale. Mr. Want's first recommendation was four ounces of the fresh root sliced to half a pint of proof spirit.

Before I conclude, I must direct your attention to the observations of Dr. Maclagan, lately published in the *Monthly Journal of Medical Science*, Edinburgh, and from which your own valuable Journal of this month has an extract. I observe that Dr. Maclagan states that the flowers of colchicum are supposed to be the part of the plant used in the preparation of the Eau Medicinale.—*London Pharm. Jour., April* 1852.

